# 4110 Frequency and patterns of polysubstance use among adults: Findings from a Focus Group that guide development of rodent models for translational research

**DOI:** 10.1017/cts.2020.393

**Published:** 2020-07-29

**Authors:** Deepthi S Varma, Yiyang Liu, Catherine Striley, Barry Setlow, Lori Knackstedt, Linda B. Cottler

**Affiliations:** 1 University of Florida Clinical and Translational Science Institute; 2 University of Florida

## Abstract

OBJECTIVES/GOALS: To explore the patterns, sequence, quantity, frequency and duration of poly substance use among adults for back translation of information to rodent models. METHODS/STUDY POPULATION: From May –December 2019, we conducted 13 focus group discussions with adults 19 to 63 years of age who reported concurrent use of cocaine with alcohol and/or marijuana in the past 30 days. All participants were recruited from the community through community outreach activities. Written informed consent was obtained and all focus group discussions were audio recorded, transcribed and analyzed using the qualitative data analysis software Atlas Ti™. RESULTS/ANTICIPATED RESULTS: A total of 34 cocaine users, (68% male, and 59% minority) participated. The majority reported cocaine as the drug of preference, while marijuana and alcohol were used to extend or control the *‘highs’*, or *‘to take the edge off’* after cocaine use. All participants reported when they used alcohol with cocaine, they could keep drinking a large amount of alcohol without feeling its effect. Participants also reported using marijuana throughout the day while driving, at work, or in class. Frequent patterns noted for the study included using two drugs at the same time or right before or after each other with alcohol used throughout the day. Participants also gave feedback on our Poly Substance Use (PSU) assessment that captures exact patterns so that the most common can be translated for the rodent models. DISCUSSION/SIGNIFICANCE OF IMPACT: Our focus group discussions provided detailed information on patterns, sequence, quantity, and types of poly substance use that could be useful for developing a poly substance use assessment in the collection of data for rodent models to understand effects of poly substance use.

